# Protective Effects of Bogijetong Decoction and Its Selected Formula on Neuropathic Insults in Streptozotocin-Induced Diabetic Animals

**DOI:** 10.1155/2017/4296318

**Published:** 2017-08-16

**Authors:** Ki-Joong Kim, Uk Namgung, Chung Sik Cho

**Affiliations:** Department of Oriental Medicine, Daejeon University, Daejeon 300-716, Republic of Korea

## Abstract

Bogijetong decoction (BGJTD) is a mixture of herbal formulation which is used in the traditional Korean medicine for the treatment of neuropathic pain caused by diabetes. Here, we investigated the regulatory effects of BGJTD and its reconstituted decoction subgroups on the neuropathic responses in streptozotocin- (STZ-) induced diabetic animals. Be decoction (BeD) was formulated by selecting individual herbal components that induced neurite outgrowth most efficiently in each subgroup. BeD induced the neurite outgrowth in DRG neurons most efficiently among decoction subgroups and downregulated the production of TNF-*α* from the sciatic nerves in STZ-diabetic animals. While the levels of phospho-Erk1/2 were elevated in the sciatic nerves of STZ-diabetic animals by BGJTD and BeD treatments, p38 level was downregulated by BGJTD and BeD. A single herbal component of BeD induced neurite outgrowth comparable to BeD and was involved in the regulation of Erk1/2 activation and TNF-*α* production in DRG neurons. Oral administration of BGJTD and BeD in STZ-diabetic animals reduced the latency time responding to thermal stimulation. Our results suggest that the reconstituted formulation is as effective as conventional BGJTD in inducing biochemical and behavioral recoveries from the neuropathy in peripheral nerves and thus the experimental reductionism may be applied to develop the methodology for compositional analysis of herbal decoctions.

## 1. Background

Diabetes is a disease causing high mortality and morbidity with complications and represents 5% of prevalence worldwide and 12–14% of prevalence in the United States in 2011-2012 [1]. Diabetes shows clinical presentation in major body organs such as kidneys, heart, pancreas, and eyes and thus results in diverse pathological symptoms and functional disturbances.

Of several animal models that have been developed to study type 1 and type 2 diabetes, streptozotocin- (STZ-) induced diabetic model is widely used because the experimental procedure is simple to induce hyperglycemia in studying type 1 diabetes and the procedure can also be used as an animal model for type 2 diabetes by feeding with high fat diet [2]. STZ, a glucosamine-nitrosourea compound, is transported preferentially into the pancreatic cells through the glucose transporter 2 (GLU2), in which it results in DNA damage that leads to the inhibition of insulin production [3]. Hyperglycemia is easily induced within several days after STZ injection and maintained for several months, which is thus useful to investigate diabetes-related complications such as peripheral neuropathy. Hyperglycemia causes metabolic disturbances such as unregulated increases in sorbitol and fructose as well as glucose in the cytoplasm of affected cells and increases osmotic stress to cells, causing the induction of signaling pathways such as MAP kinase pathway [4]. Antidepressants such as tricyclic compounds and SSRIs and anticonvulsants are used to alleviate diabetic neuropathic pain [5, 6]. In the peripheral nerves, physiological functions of Schwann cells and axons might be affected in STZ-diabetic animals, but the underlying mechanisms on cellular responses remain to be investigated.

BGJTD is the herbal formulation which was developed to treat peripheral neuropathy as sequelae of diabetes [7]. Previously, we reported that BGJTD administration into rodents was effective in attenuating the peripheral nerve damage caused by taxol injection and crush injury to the sciatic nerve [8]. In STZ-diabetic animal model, oral administration of BGJTD improved the regenerative responses of sciatic nerve axons in terms of upregulation of the marker proteins of axonal regeneration such as axonal growth-associated protein 43 (GAP-43), cell division cycle 2 (Cdc2), and phospho-vimentin as well as distal elongation of regenerating axons [9]. Moreover, some of herbal components of BGJTD revealed protective effects on tissues and organs in STZ-diabetic animals. Notably, antioxidant activity and antiacetylcholinesterase activity of* Chaenomeles sinensis* [10], attenuating effects of the extract of* Pueraria thunbergiana* on diabetic nephropathy [11], and protective effects of* Panax ginseng* on hippocampal neurons, kidneys from nephropathy, and pancreatic cells [12–14] were demonstrated in STZ-diabetic animal models.

While the previous studies indicate the potential efficacy of BGJTD to protect peripheral nerves from neuropathic insults, its compositional diversity imposes an obstacle to analytical studies because BGJTD is composed of as many as 18 different herbal components. Thus, we have begun our study by dividing BGJTD into 4 different decoction subgroups based on the traditional medicinal theory, constituted a new formulation composed of active herbal components selected from 4 subgroups, and investigated the effects on peripheral nerve responses in STZ-diabetic rodents. Our data show that a new formula is as effective as BGJTD in mediating the recovery of the peripheral nerves from neuropathic insults.

## 2. Materials and Methods

### 2.1. Herbal Drug Preparation

Key features of herbal drugs used in the present study have been described in our recent paper in which the information of purchase and quality control of BGJTD and the validation of chemical ingredients were specified in detail [8]. In the present study, 18 different herbal components of BYHWD were divided into 4 subgroups Ba, Bb, Bc, and Bd based on the theoretical description of individual ingredients. The major consideration for subgrouping was flow of qi energy, holistic balance of yin and yang spirit in the body, and blood flow and pain control, as described in our previous study [8]. Herbal compositions of each subgroup decoction are listed below (scientific names and, in parenthesis, their abbreviations and herbal names). Ba decoction was composed with* Astragalus membranaceus (AM; Astragali Radix)*,* Panax ginseng C. A. Meyer* (PG; Ginseng Radix),* Epimedium koreanum *Nakai (EK; Epimedii Herba), and* Ciborium barometz* J. Smith (CB; Cibotii Rhizoma) (weight ratio of 30 : 4 : 10 : 10); Bb with* Angelica gigas* (AG; Angelicae Gigantis Radix),* Rehmannia glutinosa* (RG; Rehmanniae Radix Preparat),* Cnidium officinale* Makino (CO; Cnidii Rhizoma), and* Spatholobus suberectus *Dunn (SS; Spatholobi Caulis) (weight ratio of 7.5 : 10 : 7.5 : 12); Bc with* Prunus persica* (PP; Persicae Semen),* Paeonia lactiflora* Pall (PL; Paeoniae Radix Rubra),* Carthamus tinctorius* (CT; Carthami Flos), Lumbricidae (LB;* Lumbricus*), and* Salvia miltiorrhiza* (SM; Salviae Miltiorrhizae Radix) (weight ratio of 7.5 : 7.5 : 7.5 : 5 : 12), and Bd with* Uncaria rhynchophylla* (UR; Uncariae Ramulus et Uncus),* Pueraria lobata *Ohwi (PL; Puerariae Radix),* Crassostrea gigas* (CG;* Crassostrea gigas*),* Albizia julibrissin *Durazz (AJ; Albiziae Cortex), and* Chaenomeles sinensis *Koehne (CS; Chaenomelis Fructus) (weight ratio of 12 : 8 : 12 : 12 : 8). Preparation of BGJTD and subgroup decoctions was essentially the same as described in our previous report [8], and final products in powders were stored at −20°C or diluted in saline solution. Animals were treated with herbal drugs by oral administration (400 mg/kg, 0.1 and 0.5 ml injection for mice and rats, resp.) on a daily basis for 2 weeks. Optimal dose was determined by analyzing dose response to injected BGJTD in STZ-diabetic mice (see below).

### 2.2. Experimental Animals and Preparation of Diabetic Animals

**Figure 1:**
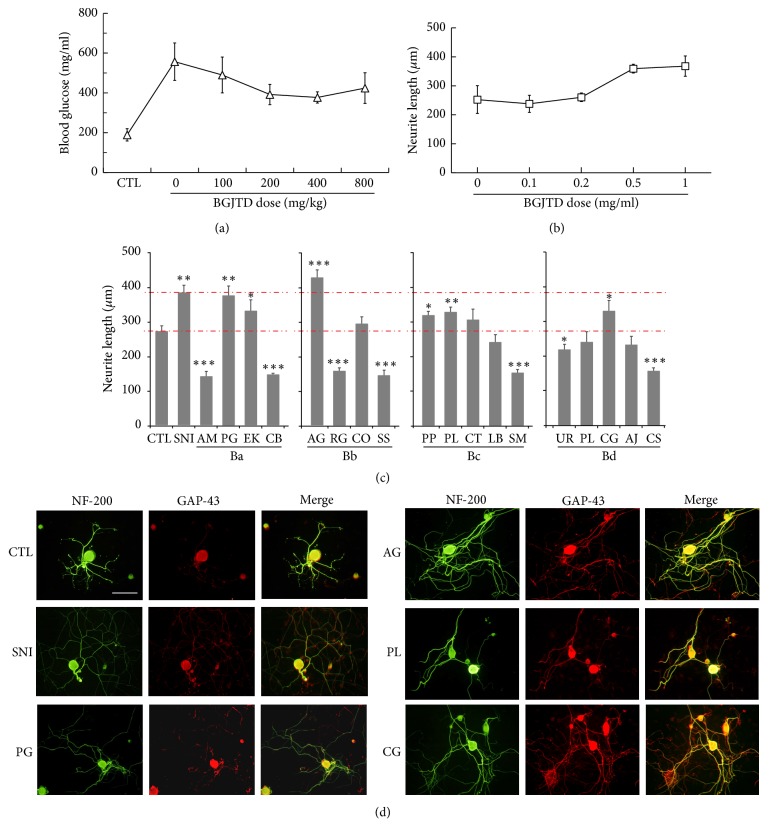
Dose responses to BGJTD and comparison of neurite outgrowth in DRG neurons after the treatment of individual constituents of BGJTD. (a) Changes of blood glucose levels with increasing doses of BGJTD. DGJTD up to 800 mg/kg was administered for 7 days to STZ-diabetic mice. Number of animals per group = 4. (b) Measurement of neurite length of DRG neurons in culture after the treatment of BGJTD. DRG neurons prepared from rats were cultured for 48 h in the presence of BGJTD at 0–1.0 mg/ml, and neurite length was measured. Number of independent experiments = 4. (c) Comparison of neurite outgrowth in individual subgroups Ba–Bd. Upper and lower red dotted lines denote the values of neurite length from animals given preconditioning nerve injury (SNI) and untreated control (CTL), respectively. Mean values of neurite length were compared among experimental groups (number of independent experiments = 4). ^*∗*^*p* < 0.05, ^*∗∗*^*p* < 0.01, ^*∗∗∗*^*p* < 0.001 versus untreated control (CTL). Unabbreviated names of all herbal drugs are described in Materials and Methods. (d) Representative fluorescence images of DRG neurons treated with herbal drugs scoring the highest neurite extension from each subgroup, along with CTL and SNI groups. In (c) and (d), herbal drugs (0.5 mg/ml) were treated to DRG neurons for 48 h and harvested for immunofluorescence staining for NF-200 (green) and GAP-43 (red) signals. Scale bar in (d) = 100 *μ*m.

We used Sprague-Dawley rats (male, 200–250 g) and Balb/c mice (male, 20–25 g), which were purchased from Samtago (Seoul, Korea). They were maintained in animal rooms which were regulated at constant temperature (22°C) and humidity (60%) with a day and night cycle (lights on 7 am to 7 pm). We strictly observed the animal handling protocols regarding “the Animal-use Statement and Ethics Committee Approval Statement for Animal Experiments provided by Daejeon University (Daejeon, Korea)” as in our previous study [15]. STZ (Sigma, St. Louis, USA) was ip injected (60 mg/kg for mouse and 45–50 mg/kg for rat) and blood glucose levels were measured by using a glucose meter (Accu-Chek, Roche Diagnostic, GmbH, Germany) before and after STZ injection and every 7 days up to 7 weeks. Animals showing higher than 300 mg/dl (or 3 mg/ml) of blood glucose at 1 week or 4 weeks after STZ injection were selected as diabetes-induced animals and used for the experiments. To determine optimal dose of herbal drug administration, mice were injected with STZ (60 mg/kg), and, 3 days later, animals showing 3 mg/ml of blood glucose or higher were selected and treated with different doses of BGJTD. Blood glucose levels were measured 7 days later. As shown in Figure 1(a), 400 mg/kg of BGJTD was the most effective in regulating glucose levels. We applied a dose of 400 mg/kg to other herbal drugs as well as BGJTD and used it for the rest of the present study.

### 2.3. Primary DRG Neuron Culture

Primary neurons were isolated from dorsal root ganglion (DRG) at lumbar levels 4 and 5 in adult rats. We prepared primary DRG neurons as described in our previous report [16]. Briefly, cells were dissociated and plated on the coverslips (Bellco, Glass Inc., Vineland, USA) which had been treated with solution of 0.01% poly-L-ornithine (Sigma) and laminin (0.02 mg/ml, Collaborative Research, Bedford, USA) for overnight. Cells were cultured in Dulbecco's Modified Eagle's medium (DMEM) containing 5% fetal bovine serum (GIBCO, Australia), 5% horse serum, 2 mM glutamine, and 1% penicillin-streptomycin and incubated for 48 hours before the harvest for immunofluorescence staining. Primary antibodies used for immunofluorescence staining were anti-neurofilament 200 (NF-200, mouse monoclonal, rabbit polyclonal, 1 : 400, Sigma), anti-TNF-*α* (1 : 400, Rabbit polyclonal, Sigma), anti-phospho-Erk1/2 (1 : 400, rabbit polyclonal, Cell Signaling Tech, Boston, USA), and anti-GAP-43 (1 : 400, rabbit polyclonal, Santa Cruz Biotech. Dallas, USA) antibodies, and secondary antibodies were fluorescein-goat anti-mouse (1 : 400, Molecular Probes, Eugene, USA) and rhodamine-goat anti-rabbit antibodies (1 : 400, Invitrogen, Carlsbad, USA). Immunofluorescence images of cultured cells were analyzed by using fluorescence microscope and the digital images were captured and transferred to the Adobe Photoshop program (version 7.0). Length of neurites was measured by using i-Solution software program (Image and Microscope Technology, Goleta, USA). At least 20 neurons were randomly selected from the microscopic field and average length of neurites in each cell was determined. To determine optimal dose of herbal drugs in culture, DRG neurons were treated with different doses of BGJTD (0.1–1.0 mg/ml) and incubated for 48 hours before the harvest for immunofluorescence staining with anti-NF-200 antibody. Measurement of neurite length revealed that a concentration of 0.5 mg/ml of BGJTD was the most optimal (Figure 1(b)), and thus this dose was used for in vitro analyses of herbal drugs for the rest of present investigation.

### 2.4. Western Blot Analysis

Proteins expressed in the sciatic nerve were determined by western blot analysis. Sciatic nerves were rapidly dissected from rats and used for protein analysis. All the procedures including protein extraction, SDS-polyacrylamide gel electrophoresis (SDS-PAGE), and western blotting were essentially the same as described in our previous report [8]. Briefly, cells in the sciatic nerves were lysed using triton lysis buffer, sonicated, and centrifuged to collect the supernatant containing proteins. Protein (15 *μ*g) was used for SDS-PAGE (10%) and western blotting analyses. Anti-phospho-Erk1/2 (1 : 1250, rabbit polyclonal, Cell Signaling, Seattle, USA), anti-p38 (1 : 1250, rabbit polyclonal, Santa Cruz Biotech.), anti-TNF-*α* (1 : 1250, rabbit polyclonal, Sigma), and anti-actin (1 : 10,000, mouse monoclonal, MP Biomedicals, Santa Ana, USA) antibodies were used as primary antibodies, and horseradish peroxidase- (HRP-) conjugated antibody (1 : 1250; goat anti-rabbit or goat anti-mouse, Santa Cruz Biotech.) as a secondary antibody. Protein band intensity in the scanned images of X-ray film was determined by using the i-Solution software (Image & Microscope Technology, Daejeon, Korea).

### 2.5. Immunofluorescence Staining of Nerve Sections

After in vivo experiments, sciatic nerves were rapidly isolated from rats and the longitudinal or transverse nerve sections (20 *μ*m) were prepared by using a cryostat (Leica CM1850, Germany). All the immunohistological procedures including fixation and permeabilization of sections, blocking, and primary and secondary antibody reactions were performed as previously described [8]. Primary antibodies used for immunofluorescence staining of nerve sections were anti-NF-200 (mouse monoclonal, 1 : 400, Sigma, St. Louis), anti-phospho-Erk1/2 (1 : 400, rabbit polyclonal, Cell Signaling), anti-p38 (1 : 400, rabbit polyclonal, Santa Cruz Biotech.), and anti-TNF-*α* (1 : 400, rabbit polyclonal) antibodies, and secondary antibodies were fluorescein-goat anti-mouse (1 : 400, rabbit polyclonal, Molecular Probes, Eugene, USA) and rhodamine-goat anti-rabbit (1 : 400, Invitrogen, Carlsbad, USA) antibodies. The nuclei in the nerve sections were visualized by staining with Hoechst dye 33258 (2.5 *μ*g/ml) (bisbenzimide; Sigma). Immunofluorescence images in the sections were analyzed by using a Nikon fluorescence microscope, and digital images were transferred to the Adobe Photoshop program. For some analytical purpose, immunofluorescence images of two different colors were merged by using layer-blending options in the Adobe Photoshop program.

### 2.6. Real-Time PCR Polymerase Chain Reaction (RT-PCR)

We extracted total RNA from DRG at lumbar levels 4 and 5 in mice and performed real-time PCR to compare the expression levels of TNF-*α* mRNA among animal groups. In our recent report, we analyzed TNF-*α* mRNA in the spleen of LPS-injected mice and found the effects of acupuncture and vagotomy on TNF-*α* mRNA expression [15]. Here, real-time PCR for DRG RNA was performed essentially the same as described in the previous investigation. Quantification of TNF-*α* mRNA level relative to GAPDH control mRNA was performed by using a software converting program (Applied Biosystems) and the data were presented as fold changes to control mRNA expression.

### 2.7. Hot Plate Test

Herbal decoctions (400 mg/kg) or saline was orally administered to STZ-induced diabetic mice and was supplemented on a daily basis for 14 days. Thermal sensitivity of animals was measured by hot plate test as described previously [8] with a slight modification. Briefly, animals were initially adapted to a change in temperature by placing on a warm surface (30°C) for 10 min and quickly transferred to a plate which was set to 50°C for 30 s. Animal's behavior on a hot plate was recorded by a video camera, and data were transferred and saved into a computer. The latency to the lifting response of the right hind paw and the frequency to withdraw two hind legs from the plate were assessed. Behavioral scores in each animal were averaged by conducting the tests three times with a 10 min interval.

### 2.8. Statistical Analysis

Results are shown as mean ± standard error of mean (SEM). Statistical analysis was evaluated by one-way ANOVA followed by Tukey's test post hoc analysis (SPSS computer software version 21.0), and statistical significance was reported as *p* < 0.05, *p* < 0.01, and *p* < 0.001.

## 3. Results

**Figure 2:**
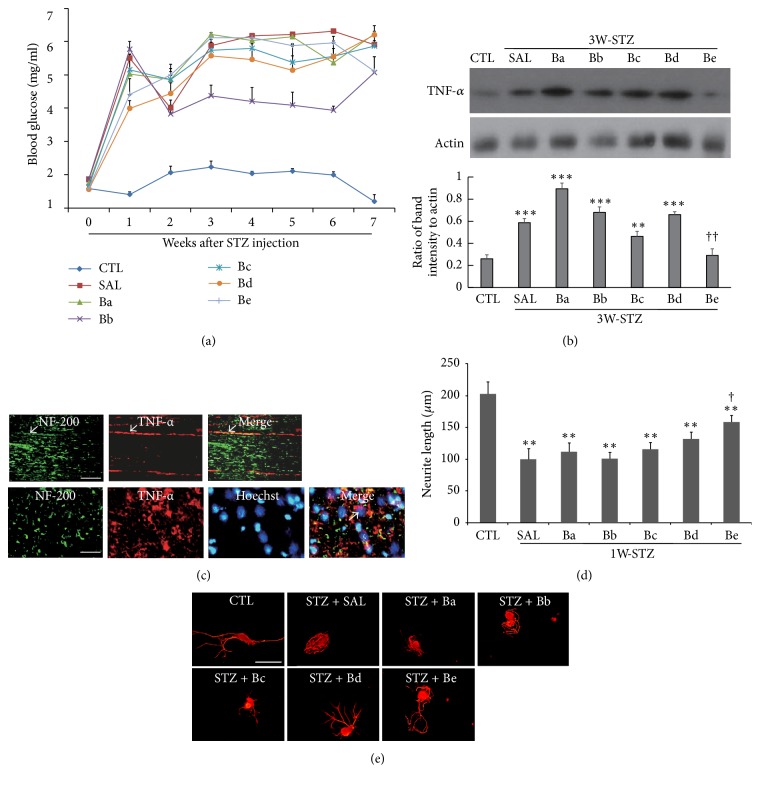
Effects of treatment of subgroup decoctions on blood glucose levels, TNF-*α* production, and neurite outgrowth. (a) A time-dependent profile of blood glucose levels in different subgroups for 7 weeks after STZ injection. Number of animals in each group = 5. (b) Western blot analysis of TNF-*α*. Images in the upper panel show the representatives from 3 independent experiments, and quantitation of protein band intensity relative to actin control is shown in the lower panel. ^*∗∗*^*p* < 0.01, ^*∗∗∗*^*p* < 0.001 versus untreated control (CTL); ^††^*p* < 0.01 versus saline control. (c) Representative immunofluorescence images showing TNF-*α* and NF-200 signals in the sciatic nerve sections prepared from STZ-diabetic animals. Arrows in the upper panel indicate TNF-*α* signals merged with NF-200-stained axons in longitudinal sections. TNF-*α* signals were also found around the Hoechst-stained nuclei (arrows in lower panel, transverse section). The sciatic nerves for western analysis in (b) were prepared from 3W-STZ animals treated with subgroup drugs as indicated in the figure, and the nerves for immunostaining in (c) were from 3W-STZ animals with saline injection. (d) Comparison of neurite outgrowth in DRG neurons prepared from STZ-diabetic animals. ^*∗∗*^*p* < 0.01 versus untreated control; ^†^*P* < 0.05 versus saline control. Number of independent experiments = 4. (e) Representative images of immunofluorescence staining of DRG neurons with NF-200. In (d) and (e), 7 days after STZ injection, DRGs at lumbar levels 4 and 5 were collected from animals showing blood glucose levels higher than 3 mg/ml and used for DRG neuron culture. Cells were treated with herbal drugs (0.5 mg/ml) for 48 h before the harvest of cells for immunofluorescence staining with NF-200 (red in (e)). Scale bars in (c) and (e) = 100 *μ*m.

For 18 different herbal components of BGJTD, we divided them into four subgroup decoctions Ba, Bb, Bc, and Bd, based on the traditional medicinal theory. We treated cultured DRG neurons with individual herbal drugs and selected 4 drugs, one from each subgroup, that induced neurite outgrowth most efficiently. Neurons treated with these selected drugs,* Panax ginseng* (PG),* Angelica gigas* (AG),* Paeonia lactiflora* (PL), and* Crassostrea gigas* (CG), showed neurite outgrowth similar to that in the DRG neurons prepared from preconditioning sciatic nerve injury (SNI) (Figure 1(c)). Furthermore, cells treated with selected drugs revealed intense GAP-43 signals in the neuritic processes as well as in the cell body (Figure 1(d)). Thus, the fifth subgroup Be decoction (BeD) composed of PG, AG, PL, and CG was used for the analysis of neural responses together with the other subgroups. Glucose levels in STZ-injected animals were maintained higher than 4 mg/ml for 7 weeks in most of animals (Figure 2(a)). In some animal groups, glucose levels were elevated 1 week after STZ injection, decreased 2 weeks later, and then steadily increased up to 7-week time point. Glucose levels in BeD subgroup, unlike other subgroups, showed a tendency of decrease between 3 and 7 weeks.

It was reported that STZ treatment in vivo and in vitro evokes inflammatory responses as identified by the induction of inflammatory cytokines in the peripheral nerves [17]. Here we injected rats with STZ and, 1 week later, treated rats with herbal drugs for 2 weeks (3W-STZ group). STZ injection significantly increased TNF-*α* in the sciatic nerve, and the protein band intensity was decreased most dramatically by BeD treatment among the subgroup decoctions (Figure 2(b)). Immunofluorescence analysis showed that TNF-*α* signals in 3W-STZ animals were colocalized with NF-200 signals, indicating the presence of TNF-*α* in axons (Figure 2(c), arrows in the upper panel). TNF-*α* signals were also found in the regions which are in close contact with Hoechst-stained nuclei, suggesting the production of TNF-*α* from nonneuronal cells such as Schwann cells. (Figure 2(c), arrow in the lower panel). To examine the effects of subgroup decoctions on the neurite outgrowth, animals were injected with STZ, and DRGs were prepared from diabetes-induced rats 7 days later. Length of neurite was significantly shorter in DRG neurons from diabetic animals compared to those from untreated control (Figure 2(d)). Treatment of BeD significantly improved the neurite outgrowth while the other decoctions were little or slightly effective (Figures 2(d) and 2(e)). Taken together, these data suggest that a newly formulated BeD may protect the peripheral nerves from inflammation in STZ-diabetic animals and facilitate axonal growth.

**Figure 3:**
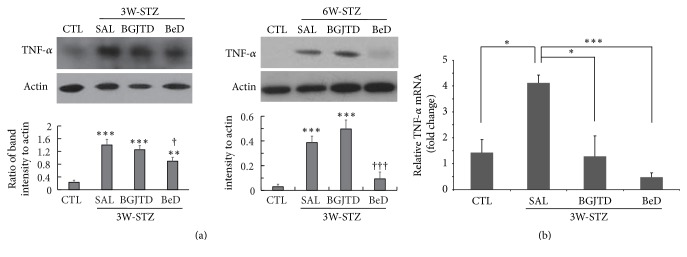
Comparison of TNF-*α* levels in the sciatic nerve of STZ-diabetic animals after herbal drug treatments. (a) Western blot analysis of TNF-*α* in the sciatic nerves from 3W-STZ and 6W-STZ animal groups. Animals were injected with STZ and, 1 week and 4 weeks later, subjected to daily administration of herbal drugs for 2 weeks (labeled 3W-STZ and 6W-STZ, resp.). Images in the upper panel show the representatives from 3 independent experiments, and quantitation of protein band intensity relative to actin control is shown in the lower panel. ^*∗∗*^*p* < 0.01, ^*∗∗∗*^*p* < 0.001 versus untreated control (CTL); ^†^*p* < 0.05, ^†††^*p* < 0.001 versus saline control. (b) Real-time PCR for the comparison of TNF-*α* mRNA expression in the DRG neurons among experimental groups. ^*∗*^*p* < 0.05, ^*∗∗∗*^*p* < 0.001. Number of independent experiments = 4.

Then, we investigated whether BeD was comparable to BGJTD in regulating neural responses in STZ-diabetic animals. Here in addition to 3W-STZ group, we prepared 6W-STZ animals in which BGJTD and BeD were administered for 2 weeks following a 4-week interval after STZ injection. In 3W-STZ animals, TNF-*α* production, which was induced by STZ injection, was slightly decreased by BGJTD and further decreased by BeD treatments (left panel in Figure 3(a)). Interestingly in 6W-STZ animals, TNF-*α* production was greatly suppressed by BeD (right panel in Figure 3(a)). To examine whether TNF-*α* production in the sciatic nerve was regulated at gene expression level, we analyzed mRNA expression by real-time PCR in the DRG at lumbar levels 4 and 5 where the cell bodies of sciatic sensory neurons are located. TNF-*α* mRNA levels in the DRG were significantly elevated in 3W-STZ rats compared to untreated control animals and then significantly downregulated by the administration of BGJTD and BeD (Figure 3(b)).

**Figure 4:**
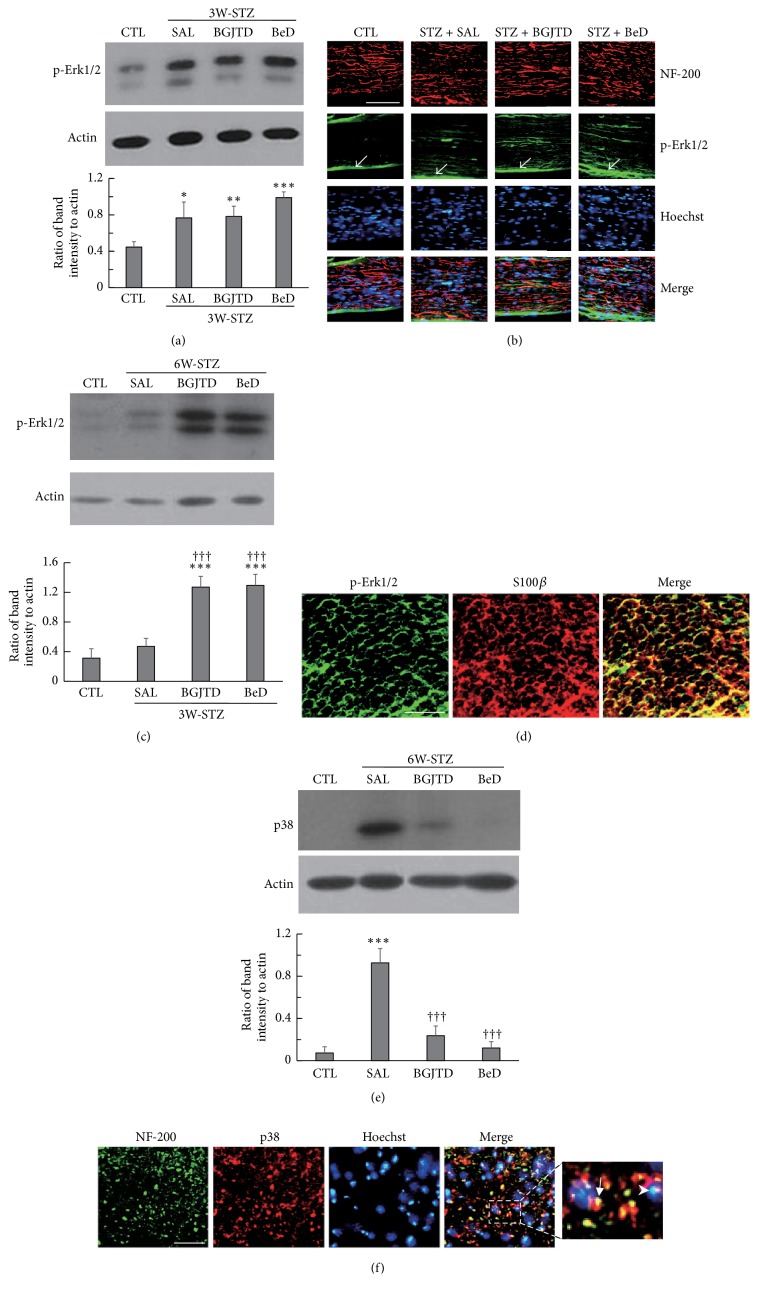
Induction pattern of phospho-Erk1/2 and p38 proteins in the sciatic nerve of STZ-diabetic animals after herbal drug treatments. Animals were injected with STZ and, 1 week and 4 weeks later, subjected to daily administration of herbal drugs for 2 weeks (labeled 3W-STZ and 6W-STZ respectively). (a) Western blot analysis of phospho-Erk1/2 in the sciatic nerve from 3W-STZ animal group. (b) Immunofluorescence images showing phospho-Erk1/2 signals in NF-200-stained sciatic nerve sections, which were prepared from BeD-treated 3W-STZ animals. Phospho-ERK1/2 signals in the epineurial sheath are indicated by arrows. (c) Western blot analysis of phospho-Erk1/2 in the sciatic nerve from 6W-STZ animal group. (d) Immunofluorescence images showing phospho-Erk1/2 and S100*β* signals in the transverse nerve section, which were prepared from BeD-treated 6W-STZ animals. (e) Western blot analysis of p38 in the sciatic nerve from 6W-STZ animal group. (f) Immunofluorescence images showing p38 and NF-200 signals in the transverse nerve section, which were prepared from saline-treated 6W-STZ animals. Western blotting images in (a), (c), and (e) are the representatives from 3 independent experiments (upper panels), and quantitation of protein band intensity relative to actin control is shown in the lower panels. ^*∗*^*p* < 0.01, ^*∗∗*^*p* < 0.01, ^*∗∗∗*^*p* < 0.001 versus untreated control (CTL); ^†††^*p* < 0.001 versus saline control. Scale bars in (b), (d), and (f) = 100 *μ*m.

Elevated levels of blood glucose can induce signaling events such as the activation of MAP kinase pathway in the target cells by acting as osmotic and oxidative stressors [18]. We examined whether the treatment of BGJTD and BeD was involved in regulating phospho-Erk1/2 and p38 kinase levels in STZ-diabetic animals. Phospho-Erk1/2 level was elevated in the sciatic nerves in 3W-STZ animals compared with untreated control, and slightly upregulated by BeD treatment without showing statistical significance (Figure 4(a)). Histological examination of the sciatic nerves in 3W-STZ animal revealed that some, but not all, of induced phospho-Erk1/2 signals were colocalized with NF-200-labeled axons (Figure 4(b)), suggesting that phospho-Erk1/2 signals were present in both axons and nonneuronal cells such as Schwann cells. It was further noted that some phospho-Erk1/2 signals were observed from the epineurial sheath (arrows in Figure 4(b)), which may contribute to phospho-Erk1/2 band intensity to some extent in the control and experimental groups in the western blot data of Figure 4(a). In 6W-STZ animals, phospho-Erk1/2 levels were slightly increased by STZ injection and then were highly upregulated by BGJTD and BeD treatments (Figure 4(c)). Phospho-Erk1/2 signals were mostly colocalized with S100*β*-labeled Schwann cells in BeD-treated STZ animals (Figure 4(d)). It was reported that Erk1/2 and p38 kinases play an opposing role in apoptosis of PC12 cells [19]. Having found that BGJTD and BeD strongly activated Erk1/2 in 6W-STZ animals, we investigated their regulatory effects on p38 production in the same animal groups. P38 was strongly induced by STZ and almost completely abolished by BGJTD and BeD treatments (Figure 4(e)), which was regulated in an opposite way to phospho-Erk1/2. In the sciatic nerve, some p38 signals were colocalized with NF-200-stained axons (arrow in Figure 4(f)) while others were seen around the Hoechst-stained nuclei (arrowhead in Figure 4(f)), indicating the production of p38 in both axons and nonneuronal cells such as Schwann cells.

**Figure 5:**
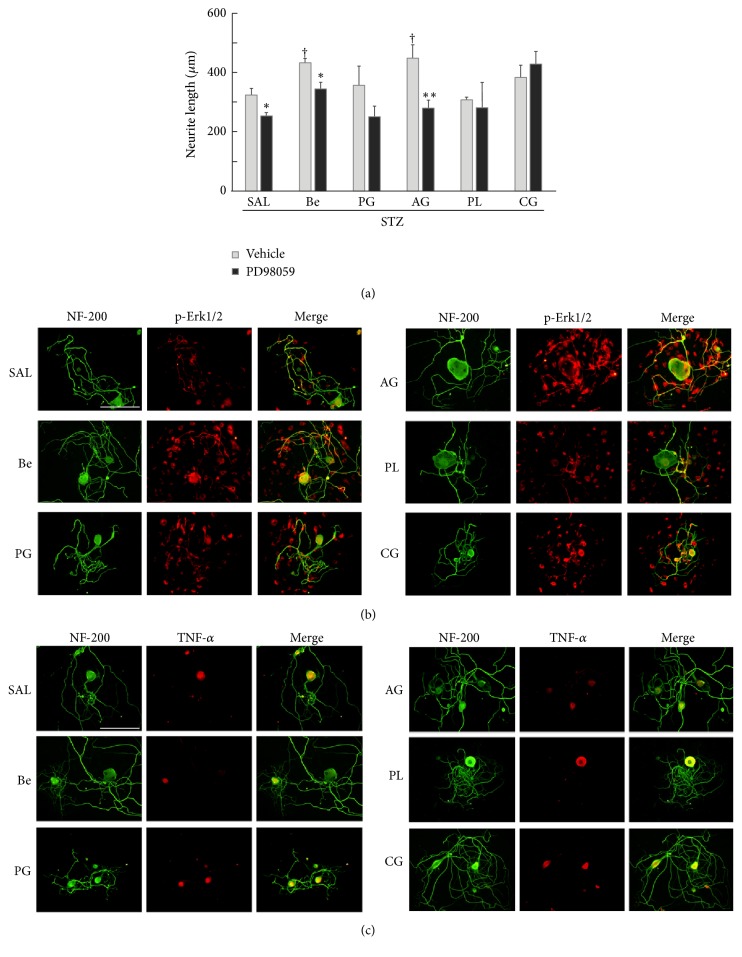
Effects of BeD herbal components on neurite outgrowth and the production of phospho-Erk1/2 and TNF-*α* in DRG neurons. (a) Comparison of neurite outgrowth in DRG neurons after treatment of herbal drugs. Cells were treated with PD98059 (10 *μ*M) or DMSO vehicle for 4 h before the addition of herbal drugs and cultured for 48 h. ^*∗*^*p* < 0.05, ^*∗∗*^*p* < 0.01 versus corresponding DMSO vehicle-treated groups; ^†^*p* < 0.05 versus saline control. Number of independent experiments = 4. (b). Immunofluorescence views of NF-200 and phospho-Erk1/2 signals in DRG neurons. (c) Immunofluorescence views of NF-200 and TNF-*α* signals in DRG neurons. In (a)–(c), DRG was prepared from STZ-diabetic rats, and cultured cells were treated with herbal drugs (0.5 mg/ml) for 48 h before cell harvest for immunofluorescence staining. Scale bars in (b) and (c) = 100 *μ*m.

Since we found that BeD showed the neuroprotective effects comparable to BGJTD in STZ-diabetic animals, we then asked whether individual constituents of BeD might have a similar activity. Treatment of BeD and AG significantly increased neurite outgrowth compared to saline control, showing a similar level of enhancement between them (Figure 5(a)). Then, treatment of MEK1/2 inhibitor PD98059 attenuated neurite growth most efficiently in neurons treated with AG drug. Phospho-Erk1/2 signals were increased in neurons and nonneuronal cells in culture by treatment of some drugs, showing strong signals in BeD- and AG-treated cells. It was noted that the cells bearing strong phospho-Erk1/2 signal revealed more intense processes in neurite outgrowth (Figure 5(b)). We also analyzed whether TNF-*α* production in DRG neurons was affected by treatment of BeD herbal components. As shown in Figure 5(c), TNF-*α* was found only in neuronal cell body, and its level was relatively lower in BeD- and AG-treated cells. Taken together, AG, a herbal component of BeD, may induce neurite outgrowth efficiently by regulating Erk1/2 activity and TNF-*α* production in DRG neurons.

**Figure 6:**
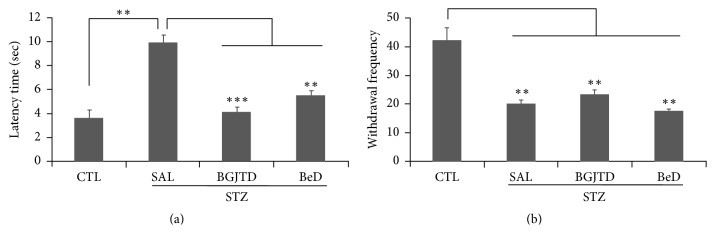
Effects of BGJTD and BeD on the heat sensitivity. STZ-diabetic mice were orally given BGJTD and BeD for 2 weeks and were subjected to the measurement of the lifting frequency of the hind paws (a) and the latency period after placing on the hot plate (b). ^*∗∗*^*p* < 0.01; ^*∗∗∗*^*p* < 0.001. Number of animals = 4.

We then examined the effects of BGJTD and BeD on the thermal sensitivity in the hind limbs in mice. The latency time for withdrawal responses on the hot plate was greatly increased by STZ injection and was significantly reduced by BGJTD and BeD treatments (Figure 6(a)). A withdrawal frequency from the hot plate was significantly decreased in STZ-treated animals compared to untreated control animals. Treatment of BGJTD and BeD failed to show significant changes in withdrawal frequency in STZ-treated group (Figure 6(b)).

## 4. Discussion

BGJTD formulation has been developed and used therapeutically in the traditional Korean medicine to alleviate neuropathy associated with diabetes [7]. To understand the biological basis on its efficacy, previously we studied the neural responses in animals given taxol and crush injury to the sciatic nerves and found the protective activity of BGJTD in the injured nerves [8]. Here using biochemical, histological, and behavioral methods, we found that BGJTD, together with an additionally formulated decoction selected from BGJTD, was effective in protecting the peripheral nerves from neuropathic insults in STZ-diabetic animals.

BGJTD is a mixture of herbal drugs composed of 18 different components. Measurement of neurite outgrowth in cultured neurons is a convenient way to examine the effects of candidate drugs on the regenerative responses of neurons given injury [20]. Previous studies also showed that the intrinsic capacity of axonal regeneration after nerve injury and neurite outgrowth are retarded in STZ-induced diabetic animals [21, 22], which may reflect the pathological impairment of axonal function such as increases in inhibitory factors of axonal regeneration and downregulation of proinflammatory cytokines [23, 24]. Thus, as an initial step to select active herbal components, we treated DRG neurons with individual herbal drugs and selected the most active component from each subgroup. Drugs such as PG, AG, PL, and CG induced the neurite outgrowth most efficiently from each subgroup and thus used for constituting a new formula Be. It was previously reported that levels of TNF-*α* were elevated in the serum, brain tissue, and peripheral nerves in STZ-diabetic animals [25, 26], and its level was further upregulated in the serum by LPS injection [27]. In the present study, TNF-*α* was induced in the sciatic nerve in rats 3 weeks after STZ injection. Strong TNF-*α* signals were colocalized with NF-200-labeled nerve fibers, and additional signals were observed in the surrounding nonneuronal cells such as Schwann cells as reported previously [28]. Examination of subgroup decoctions on the regulation of inflammatory responses in STZ-diabetic animals revealed that BeD was most effective in downregulating TNF-*α* levels in the sciatic nerve of STZ-diabetic animals. Treatment of BGJTD and BeD similarly attenuated TNF-*α* mRNA expression in the DRG, suggesting that BGJTD and its active components may be involved in regulating TNF-*α* production in neuronal cells and Schwann cells at gene expression level.

Since BeD was the collection of active herbal components inducing the neurite outgrowth, we investigated whether BeD was superior to other subgroup decoctions in inducing neurite outgrowth. Neurite outgrowth was largely retarded in DRG neurons which had been prepared from STZ-diabetic rats compared control animals. These data suggest that, on the contrary of the potential effects of preconditioning injury for the enhancement of neurite growth [29], hyperglycemic environment by STZ treatment may suppress the gene expression in DRG neurons leading to attenuate axonal growth. BeD facilitated neurite outgrowth most efficiently, implying that the herbal constituents of BeD may protect DRG neurons from the neuropathic insults by STZ.

Having confirmed that BeD regulated inflammatory reactions in the sciatic nerves and promoted neurite outgrowth, we explored its effects on neural tissues in vivo. Induction levels of phospho-Erk1/2, which was measured as an indicator of activation of cells in the sciatic nerve, were different between 3W-STZ and 6-W STZ animal groups. Then, phospho-Erk1/2 was upregulated by BGJTD and BeD treatment in both groups, suggesting the activation for neural pathway for survival. In the sciatic nerves, some, but not all, of phospho-Erk1/2 signals were observed in axons. Phospho-Erk1/2 can be induced by neurotrophic signaling in the peripheral nerves given injury and retrogradely transported into the cell body where it is involved in target gene expression [30–32]. Here, we speculate that axonal phospho-Erk1/2 signals, which were initially induced by osmotic stress in STZ-diabetic animals, were further upregulated by BGJTD and BeD to activate intracellular signaling pathway in DRG neurons. In addition, phospho-Erk1/2 signals found in Schwann cells may play a role in regulating neural activity locally. In contrast to neuronal survival by Erk1/2 activation, activation of p38 and JNK pathways was known to activate apoptotic pathway in PC12 cell [19]. Indeed, our data showed that p38 protein, which was increased by STZ injection, was dramatically decreased by BGJTD and BeD treatments. We also noted that phospho-Erk1/2 and p38 signals were observed in Schwann cells in the sciatic nerve in STZ-diabetic animals. Although our results do not provide direct evidence on the regulation of these proteins in Schwann cells, it is highly likely that BGJTD and BeD act on Schwann cells, considering that Schwann cell interaction with axons is critical for the recovery from neuropathic pain and nerve injury [33, 34]. Phospho-Erk1/2 and p38 may respond to BGJTD and BeD treatments in an opposite way in the sciatic nerve axons and Schwann cells so as to protect neurons and Schwann cells from the hyperglycemic stress in diabetic animals. Future studies on the induction pattern of neurotrophic factors and inflammatory cytokines may provide insights into mechanistic basis on the signaling event mediated by BGJTD and BeD in STZ-diabetic animals.

Analysis of behavioral response showed that treatments of BDJTD and BeD reduced the latency time responding to thermal stimulation while both drugs failed to change withdrawal frequency. Decreased latency to heat may indicate the improvement of reflexive behavior to thermal stimulation and associated pain [35]. Whether these behavioral changes are linked to biochemical responses in the sciatic nerves remains to be explored.

## 5. Conclusion

Our study shows that BGJTD and BeD improved neural responses in the sciatic nerves from neuropathic insults in STZ-diabetic animals. BGJTD is a herbal formulation which has been prescribed for the treatment of diabetic neuropathic pain in clinical practice in Korea [7]. A newly formulated BeD is composed of four herbal components and they may be involved in elevating erythropoietic activity, tonifying qi and blood flow into some stagnant body parts, and replenishing yin energy while subduing yang, according to traditional medicinal theory. Consequently, augmented vital energy may alleviate pain which is caused by stagnation of qi and blood flow. Ginsenoside Rb1 isolated from PG and albiflorin and paeoniflorin both from PL have been identified as the chemical ingredients of BeD [8]. Growing body of evidence indicates that Rb1 has a protective effect on neural tissues related to inflammatory and degenerative diseases [36]. The isomers albiflorin and paeoniflorin were reported to have anti-inflammatory activity and regulate neuropathic pain in spinal neurons [37, 38]. Interestingly, a herbal recipe called Tang Luo Ning, which contains both albiflorin and paeoniflorin among others, was shown to attenuate oxidative stress in STZ-induced diabetic rats [39]. Finally, our data demonstrate that treatment of AG into the DRG neurons from STZ-diabetic animals increases neurite outgrowth and regulates the production of phospho-Erk1/2 and TNF-*α*. Therefore, it is tempting to design pharmacological studies exploring the signaling pathways mediated by chemical ingredients and herbal components of BeD. In conclusion, the present results suggest that the experimental reductionism may be applied to explore the biological basis on the efficacies of herbal drugs or decoctions.
